# Regulating astrocytic activity in the dorsal striatum mitigates L-dopa-induced dyskinesia in Parkinson’s disease

**DOI:** 10.1038/s41598-025-12104-5

**Published:** 2025-07-22

**Authors:** Young-Kyoung Ryu, Hye-Yeon Park, Ju-Eun Kim, Hyun-Hee Seo, Chul-Ho Lee, Kyoung-Shim Kim

**Affiliations:** 1 Laboratory Animal Resource Center, Korea Research Institute of Bioscience and Biotechnology (KRIBB), Daejeon, 34141 Republic of Korea; 2 Department of Otolaryngology-Head and Neck Surgery, Chonnam National University Medical School and Chonnam National University Hospital, Gwangju, 61469 Republic of Korea; 3 KRIBB School, University of Science and Technology, Daejeon, 34141 Republic of Korea

**Keywords:** Abnormal involuntary movements, Striatum, AAV-GFAP-hM3D(Gq), AAV-GFAP-hM4D(Gi), Clozapine N-oxide (CNO), 6-hydroxydopamine-induced mouse model, Neuroscience, Diseases

## Abstract

In Parkinson’s disease (PD), long-term 3,4-dihydroxy-L-phenylalanine (L-dopa) therapy leads to the development of motor complications, including L-dopa-induced dyskinesia (LID). Increased numbers of reactive astrocytes in the brains of patients with PD are a key feature of this disease. Astrocytes are involved in the development of LID; however, whether the regulation of astrocytic activity influences LID development remains unclear. Therefore, this study aimed to determine the effect of the direct modulation of glial fibrillary acidic protein (GFAP)-expressing glia on LID development during L-dopa therapy in PD using chemogenetic tools. Adeno-associated viruses (AAVs) were used to target designer receptors exclusively activated by designer drugs (DREADDs) in GFAP-expressing cells to modulate Gq- or Gi-mediated signaling and regulate astrocytic activity in the brain. AAVs were injected into the dorsal striatum, and 6-hydroxydopamine (6-OHDA) was injected into the substantia nigra of mice. Clozapine N-oxide was co-administered with L-dopa. Chemogenetic activation of astrocytes in the dopamine-depleted striatum affected the early development of LID in 6-OHDA-lesioned mice. Furthermore, astrocyte suppression through Gi-mediated DREADD reduced abnormal involuntary movement scores in mice. These results suggest that regulating astrocytic activity in the dorsal striatum could be a therapeutic option for LID in PD.

## Introduction

Parkinson’s disease (PD) is a common age-related neurodegenerative disease. Its most characteristic feature is the progressive degeneration of nigrostriatal neurons, leading to motor symptoms such as tremors, stiffness, bradykinesia, and the loss of voluntary movements^1,2^. Although 3,4-dihydroxy-L-phenylalanine (L-dopa) remains the most effective treatment, prolonged use often leads to severe abnormal involuntary movements known as L-dopa-induced dyskinesia (LID)^3^. Dividing the doses of L-dopa can delay LID onset; however, this approach has limitations, particularly in severely ill patients^4^. These limitations include insufficient control of drug efficacy and the occurrence of dyskinesia independent of drug action^5^.

Astrocytes provide structural support to neurons and act as dynamic signal transducers involved in various essential physiological processes in the brain^6,7^. Chronic exposure to stress triggers astrocyte overactivation, resulting in the release of neuroinflammatory markers^8^. Persistent damage to the surrounding neurons can cause an abnormal increase in activated astrocytes, a condition known as glial scarring or astrocytosis^9,10^. This process, characterized by hypertrophy of primary processes and increased expression of the intermediate filament protein glial fibrillary acidic protein (GFAP), is referred to as astrogliosis^10^. Glial scar formation is frequently observed in the damaged brain regions of patients and animal models of PD^11,12^. Neuroinflammatory responses resulting from astrocyte hyperactivation contribute to PD and LID development^8,13^. However, drugs with protective effects against LID can inhibit astrocytic activity in the striatum of PD models^14–16^. Notably, the number of activated astrocytes and the brain area occupied by activated astrocytes significantly increase after the onset of dyskinesis compared to the early stages of L-dopa treatment^17,18^. However, whether LID development in PD can be modulated by directly regulating astrocytic activity remains unclear.

Designer receptors exclusively activated by designer drugs (DREADDs) have emerged as powerful tools for manipulating neuronal and non-neuronal signal transduction in a cell-type-specific manner in freely moving animals^19,20^. This chemogenetic technology enables selective activation or inhibition of cellular firing using small-molecule chemical activators previously unrecognized by endogenous systems^20^. Depending on their specific downstream effector systems, G-protein-coupled receptors (GPCRs) can inhibit or excite neuronal and non-neuronal firing^21^. For example, DREADD-based muscarinic receptors, such as hM1D(Gq), hM2D(Gi), hM3D(Gq), hM4D(Gi), and hM5D(Gq), are activated by clozapine-N-oxide (CNO) and are unresponsive to acetylcholine^22^. CNO-mediated activation of these receptors can modulate neuronal activity effectively in various rodent models^19,23,24^. The aim of this study was to investigate the effect of directly modulating GFAP-expressing glia on LID development during L-dopa therapy in PD using chemogenetic tools.

## Materials and methods

### Animals

This study was conducted using 9-week-old-male C57BL/6 J mice (22–27 g) provided by the Korea Research Institute of Bioscience and Biotechnology (KRIBB). Animals were housed under specific pathogen-free conditions in a temperature (22 ± 2 °C) and humidity (50–60%) controlled environment with a 12-h light/dark cycle (lights on at 07:00). The animals had free access to sterilized food and water (2018s; Envigo Teklad, Madison, WI, USA), and food and water were replenished 1–2 times a week. The cages were filled with chopped wooden bedding, which was replaced weekly. All procedures involving animals complied with the ARRIVE guidelines^25^. All animal experiments were approved by the Institutional Animal Use and Care Committee of the Korea Research Institute of Bioscience (KRIBB-AEC-20065) and Biotechnology and were performed in accordance with the Guide for the Care and Use of Laboratory Animals published by the United States National Institutes of Health. A total of 130 mice were used. To establish a 6-hydroxydopamine (6-OHDA)-induced mouse model, a total of 120 mice were used. Of these, 47 mice were excluded based on lesion success, rotations, and viral verification.

### Experimental design

6-OHDA (#H116), desipramine hydrochloride (#D3900), L-dopa (#D9626), and benserazide hydrochloride (#B7238) were purchased from Sigma-Aldrich (St. Louis, MO, USA). These compounds were used to establish PD and LID mouse models and to evaluate the therapeutic effects of L-dopa and DREADD activation. Powdered 6-OHDA was dissolved in 0.2% ascorbic acid to a concentration of 10 μg/μL and stored at − 20 °C. 6-OHDA (10 μg/μL) was diluted in saline to a concentration of 5 μg/μL immediately before use.

**Fig. 1:**
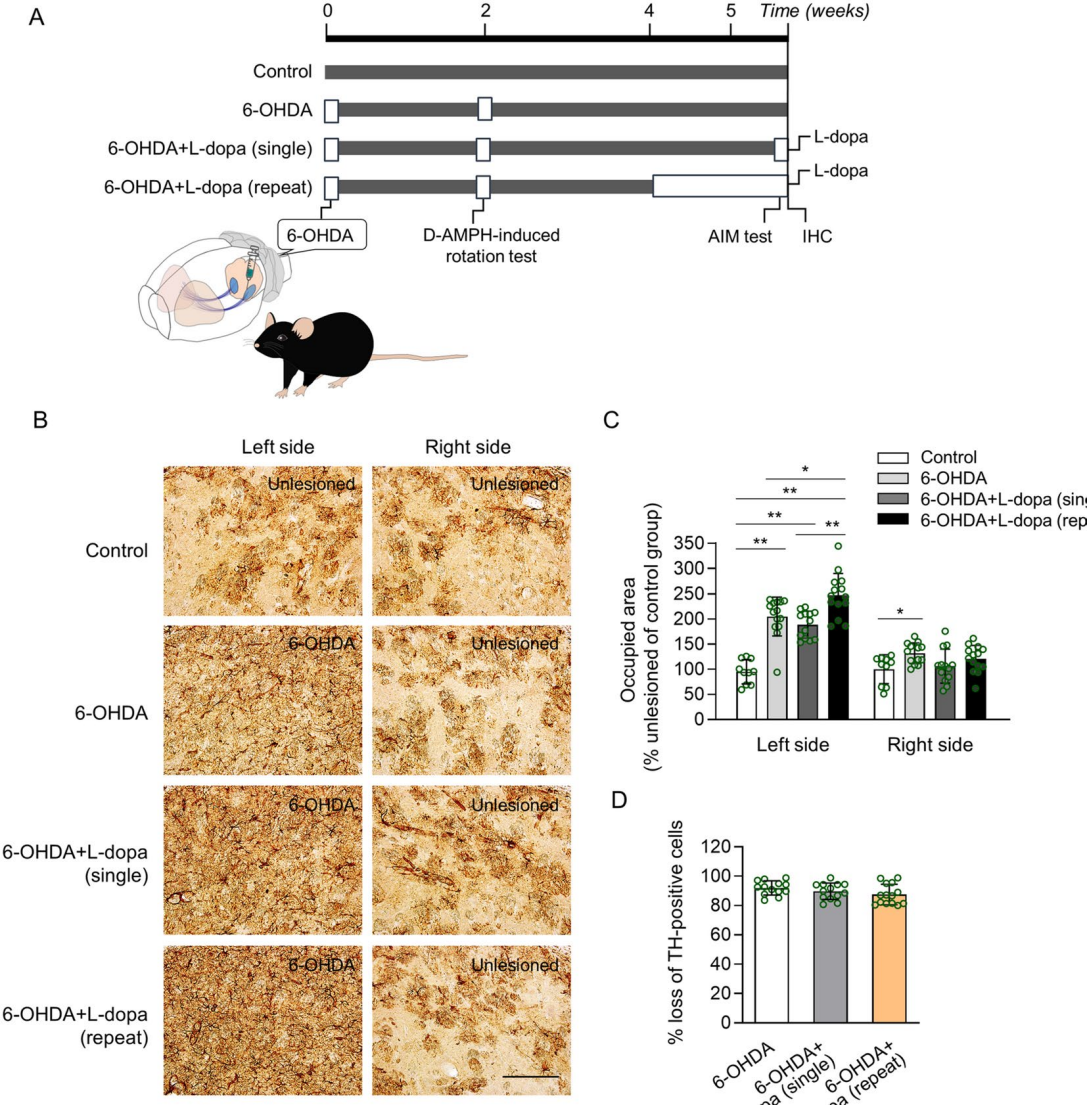
Effects of dopamine depletion and single and repetitive L-dopa administration on astrocyte activation in the striatum. (**A**) The experimental schedule of 6-OHDA, behaviour test, drug administration, and immunohistochemistry (IHC). (**B**) The immunostaining images show glial fibrillary acidic protein (GFAP)-positive astrocytes in the dorsal striatum of the control, 6-hydroxydopamine (6-OHDA), 6-OHDA/3,4-dihydroxy-L-phenylalanine (L-dopa) (single), and 6-OHDA/L-dopa (repeated) groups. Scale bar, 200 μm. (**C**) The occupied area of GFAP-positive astrocytes was analyzed in the unlesioned and 6-OHDA-lesioned side of the dorsal striatum. (**D**) Percentage loss of TH-positive cells on the lesioned side compared with those on the intact side of the left substantia nigra (SNc). Control, n = 10; 6-OHDA, n = 13; 6-OHDA/L-dopa (single), n = 13; 6-OHDA/L-dopa (repeated), n = 14. *p < 0.05, **p < 0.01 vsersu indicated group (one-way analysis of variance [ANOVA] followed by Tukey’s multiple comparisons test). Data are presented as mean ± standard deviation (SD).

To investigate the effects of dopamine depletion and L-dopa administration on astrocyte activation in the dorsal striatum, a 6-OHDA was directly injected into the substantia nigra pas compacta (SNc) (Fig. 1A). D-amphetamine-induced rotation test was performed two weeks after the 6-OHDA to determine whether 6-OHDA-induced PD motor abnormalities occurred. The mice were divided into four groups: non-treated group (Control, n = 10), 6-OHDA-lesioned group without L-dopa treatment (6-OHDA, n = 13), 6-OHDA-lesioned group with single L-dopa/benserazide treatment [6-OHDA + L-dopa (single), n = 13] and 6-OHDA-lesioned group with repeated L-dopa/benserazide treatment [6-OHDA + L-dopa (repeat), n = 14]. Control mice received no treatment and were used only for brain sampling, whereas the 6-OHDA group received 6-OHDA injections without L-dopa treatment. Four weeks after 6-OHDA injection, L-dopa was administered either as a single dose (6-OHDA + L-dopa (single) group) or repeatedly over 11 days (6-OHDA + L-dopa (repeat) group). After all experiments were completed, immunostaining of brain tissue was performed. Three independent sets of experiments were conducted, each including four experimental groups. Histological analyses were conducted concurrently.

**Fig. 2:**
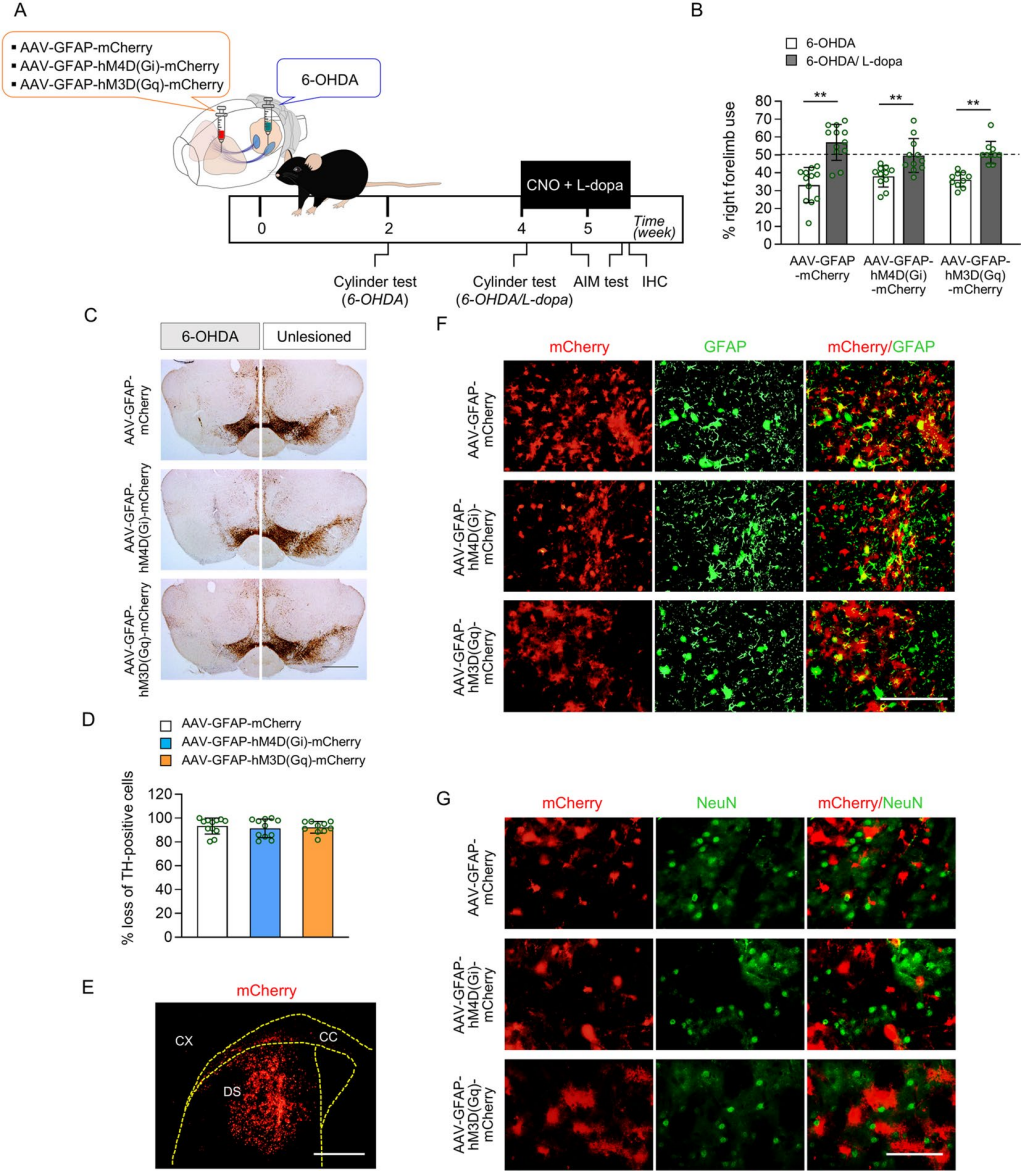
Confirmation of 6-OHDA-induced Parkinson’s disease model and AAV8-GFAP-mCherry, AAV8-hM4D(Gi)-mCherry, and AAV8-hM3D(Gq)-mCherry expression in astrocytes. (**A**) The experimental schedule of 6-OHDA and adeno-associated virus (AAV) injections, cylinder tests, abnormal involuntary movement (AIM) tests, drug administration, and immunohistochemistry (IHC). (**B**) The percentage of right forelimb use out of the total number of touches made with both paws on the cylinder in the three groups. **p < 0.01 vsersu indicated group (Student’s t-test). (**C**) The immunostaining images showed TH-positive cells in the substantia nigra of the AAV8-GFAP-mCherry, AAV8-hM4D(Gi)-mCherry, and AAV8-hM3D(Gq)-mCherry groups. Scale bar = 1 mm. (**D**) Percentage loss of TH-positive cells in the 6-OHDA-lesioned side compared with those in the intact SNc side. One-way analysis of variance [ANOVA] followed by Tukey’s multiple comparisons test. (**E**) A representative image showing the mCherry expression area in the dorsal striatum (DS) of group AAV8-GFAP-hM4D(Gi)mCherry. Scale bar = 1 mm. CC, corpus callosum; CX, cortex. (**F**) GFAP-immunostaining, AAV8-mediated mCherry expression, and their co-localization in the dorsal striatum of the mice. Scale bar = 200 μm. (**G**) NeuN-immunostaining, AAV8-mediated mCherry expression, and their co-localization in the dorsal striatum of the mice. Scale bar = 200 μm. AAV8-GFAP-mCherry, n = 12; AAV8-hM4D(Gi)-mCherry, n = 11; AAV8-hM3D(Gq)-mCherry, n = 10. Data are presented as mean ± SD.

To induce DREADD expression and dopaminergic neuronal damage, the striatum was directly injected with adeno-associated viruses (AAV)-GFAP-mCherry, AAV-GFAP-hM4D(Gi), or -AAV-hM3D(Gq), and the SNc was directly injected with 6-OHDA (Fig. 2A). The mice were divided into three groups: AAV-GFAP-mCherry (n = 12), AAV-GFAP-hM4D(Gi)-mCherry (n = 11), and AAV-GFAP-hM3D(Gq)-mCherry (n = 10) groups. A cylinder test was performed at both two and four weeks after the 6-OHDA and AAV injections to assess whether 6-OHDA-induced motor abnormalities had developed and to evaluate the therapeutic effects of L-DOPA and CNO in the PD model. L-dopa and CNO were administered simultaneously daily. The abnormal involuntary movement (AIM) test was conducted on days 5 and 10 of L-dopa and CNO administration. The brain was removed 1 h after CNO administration and 30 min after L-dopa injection on the 11th day of combined L-dopa and CNO treatment. The mice were sacrificed by quick cervical dislocation and their brain were collected and washed with phosphate-buffered saline (PBS) and fixed by soaking in 4% paraformaldehyde. Death was verified by monitoring the symptoms such as the absence of chest movement, lack of a detectable heartbeat, pale mucous membranes, no response to toe pinch and changes in eye color^26^. The study was conducted as a single set of experiments, which included all three experimental groups.

DREADD was used to modulate the function of GPCRs in striatal astrocytes. CNO (#B7238, Sigma-Aldrich), a DREADD agonist, was administered orally 30 min before the L-dopa injection. Benserazide was injected intraperitoneally 20 min after CNO, followed by intraperitoneal L-dopa injection 10 min later. The abnormal involuntary movement (AIM) test is conducted to evaluate behavioral patterns similar to those in patients with LID^27^, which was performed on days 5 and 10 after administration to investigate whether astrocyte activation affected LID development.

### AAV and 6-OHDA injection

Striatal AAV and nigral 6-OHDA injections were administered as previously described^28–30^. The mice were intraperitoneally injected with 25 mg/kg desipramine to prevent damage to noradrenergic neurons 30 min before surgery. The mice were anesthetized by intraperitoneal injection of ketamine/xylazine. Once the mice were completely anesthetized, they were fixed in a stereotaxic frame (Stoleting Europe, Dublin, Ireland), and AAV and 6-OHDA were injected into the brain. 6-OHDA (3 µL, 5 μg/μL, injection rate 1 μL/min) was directly injected into the left substantia nigra (SNc): − 3.0 mm anterior–posterior, − 1.3 mm mediolateral, and − 4.7 mm dorsoventral. AAV8 (1 μL; AAV-GFAP-mCherry, AAV-GFAP-hM3D(Gq)-mCherry, and AAV-GFAP-hM4D(Gi)-mCherry) was directly injected into the left striatum (ST): + 1.2 mm anterior–posterior, -1.8 mm mediolateral, and − 3.6 mm dorsoventral. The SNc and ST coordinates were based on the brain atlas of Paxinos and Franklin (2008).

The hM3D(Gq) coding sequence was removed from the pAAV-GFAP-hM3D(Gq)-mCherry vector (#50,478, Addgene, Watertown, MA, USA) to generate the AAV8-GFAP-mCherry control vector. The resulting AAV-GFAP-mCherry, AAV-GFAP-hM3D(Gq)-mCherry, and AAV-GFAP-hM4D(Gi)-mCherry (#50,479, Addgene) vectors of the AAV8 serotype were produced and purified using iodixanol gradient ultracentrifugation at the KIST Virus Facility (Seoul, Korea). The final viral titers were 1.4 × 10^12^ genome copies (GC)/mL for AAV8-GFAP-mCherry, 1.3 × 10^12^ GC/mL for AAV8-GFAP-hM3D(Gq)-mCherry, and 2.2 × 10^12^ GC/mL for AAV8-GFAP-hM4D(Gi)-mCherry.

Lesioned mice were intraperitoneally injected with 500 mg/kg sterile glucose–saline solution immediately after surgery and once daily for 3 days after surgery to avoid dehydration. The mice were placed in a warmer from the start of anesthesia until awakening to prevent hypothermia caused by the anesthetic. The mice were placed in their home cages for the recovery period when they awoke from anesthesia and began to move. One week after surgery, 15% sucrose solution was sprinkled on the feed and placed in a shallow container on top of the chopped wood bedding in the cage.

### Dextro-amphetamine (D-AMPH)-induced rotation test

D-AMPH-induced rotation test were conducted as described previously^28^. D-AMPH was purchased from USP (Rockville, MD, USA, 5 mg/kg, i. p.) and dissolved in saline. D-AMPH was injected into the mice, and rotation behaviours were measured in a transparent cylinder with a diameter of 20 cm and a height of 13 cm. Behaviours were recorded for 60 min and the number of ipsilateral rotations was analysed using a SMART video tracking system (Panlab, Barcelona, Spain). Mice exhibiting at least 150 rotations were selected as the primary inclusion criterion. Because the mice were selected based on subsequent cell death, the rotation count was not strictly enforced. Rotation counts were used to group animals such that the average number of rotations was comparable across groups, and mice showing more than 80% cell loss were selected for final inclusion.

### Cylinder test

Cylinder tests were conducted as described previously^28^. Behavioral experiments using the cylinder were conducted by placing one mouse in an acrylic cylinder with a diameter of 15 cm and a height of 27 cm. The behavior of the mice in the cylinder was recorded for 5 min, and the number of times both forepaws touched the cylinder wall was recorded. The number of right forepaw touches was calculated relative to the number of both forepaw touches and expressed as a percentage to determine whether the right forepaw was affected by the left SNc damage by 6-OHDA. The L-dopa-induced cylinder test was performed 1 h after CNO administration and 30 min after the L-dopa injection. No cut-off value was applied in the cylinder test. After completing all experiments, the final selected mice based on lesion success and viral verification were used to compare the effects of L-dopa treatment.

### Induction and assessment of dyskinetic movements

The AIM test was conducted as described previously^28,29,31^. Four weeks after 6-OHDA and AAV injection, mice were treated with 1 mg/kg CNO, 20 mg/kg L-dopa, and 12 mg/kg benserazide daily for 11 days. The AIMs test was conducted on days 5 and 10 of L-dopa treatment to measure the development of dyskinetic movements during LID. Immediately after the L-dopa injection, each mouse was placed in an individual glass cylinder, and its behavior was recorded. The mice were scored for dyskinetic movements for 1 min every 20 min after L-dopa treatment for 80 min. The dyskinetic movement of mice was scored according to four AIM subtypes: axial (contralateral dystonic posture of the neck and upper body towards the sides trilateral to the lesion), limb (jerky and fluttering movement softening the limb contralateral to the side of the lesion), orolingual (vacuous jaw movements and tongue protrusions), and locomotive (tight contralateral turns). The sum of the axial, limb, and orofacial subtypes represents the ALO AIMs that closely reflect the dyskinetic behavior of patients^32^. The scores ranged from 0 to 4 for each subtype, as follows: 0, absent; 1, present for less than half of the observation period; 2, present for over half of the observation period; 3, continuous but interrupted by other stimulations; and 4, continuous and uninterrupted by other stimulations.

### Immunohistochemistry

Immunohistochemistry (IHC) was performed as described previously^14,30^. The removed brains were washed once with 1 × PBS and fixed by soaking in 4% PFA for over 24 h. The fixed midbrains were sectioned into 40 μm thick coronal sections using a vibratome (Vibratome VT1000A, Leica, Germany). The tissue sections of the ST and SNc regions were washed in 1 × PBS, then immersed in storage solution and stored at − 20 °C. Sectioned tissue was placed in a 24-well plate containing 1 × Tris-buffered saline (TBS), and the storage solution was washed away to perform IHC using the free-floating method. Sectioned tissues were soaked in 3% H_2_O_2_ (v/v) solution for 10 min and then washed three times with 1 × TBS to inhibit the activity of endogenous peroxidases. The sectioned tissues were immersed in a blocking solution for 1 h at room temperature to prevent nonspecific binding of antibodies and other substances. Sectioned tissues were incubated overnight at 4 °C in a solution of 5% horse serum mixed with a tyrosine hydroxylase primary antibody (TH, 1:2000, catalog #P40101-0, Pel-Freez Biologicals, Rogers, AR, USA) and 5% goat serum mixed with a glial fibrillary acidic protein primary antibody (GFAP, 1:200, catalog #Z0334, DAKO, Glostrup, Denmark). Next, the sectioned tissues were washed thrice with 1 × TBS and incubated with biotinylated secondary anti-rabbit IgG (1:2000, Vector Laboratories, Burlingame, CA, USA) for 1 h at room temperature. After the antibody-antigen reaction, the sectioned tissues were washed with 1 × TBS and stained with 3,3′-diaminobenzidine (D5637, Sigma, Saint Louis, MO, USA) using an avidin-biotinylated peroxidase complex immunohistochemical system (ABC kit, PK-6100, Vector Laboratories). Immunofluorescence staining was performed using an Alexa Fluor 488 goat anti-rabbit IgG antibody (secondary antibody, 1:200; Life Technologies, Carlsbad, CA, USA). Sections containing the dorsal striatum were selected and incubated with anti-GFAP. TH-stained cells in the left and right SNc were counted in two sections per mouse. TH-stained cells were only counted when their nuclei were visualized in the focal plane using a MetaMorph image analyzer (Version 7.7.0.0, Molecular Devices Inc., CA, USA) to avoid double counting neurons with unusual shapes. Additionally, analysis of the area occupied by GFAP-immunoreactive astrocytes was performed using Meta Imaging Series software. To ensure consistency across all experimental groups, the intensity threshold was determined based on astrocytes located in the unlesioned (contralateral) hemisphere. Specifically, within the acquired images, the threshold was set at a level that captured not only the entire astrocytic cell bodies (somata) but also their extending processes, allowing for a comprehensive assessment of astrocytic morphology and activation. This standardized threshold was then uniformly applied to all analyzed images to minimize variability and enable reliable comparisons of GFAP expression across experimental conditions. Quantitative evaluation of immunoreactive cells was performed blinded.

### Statistical analyses

**Fig. 3:**
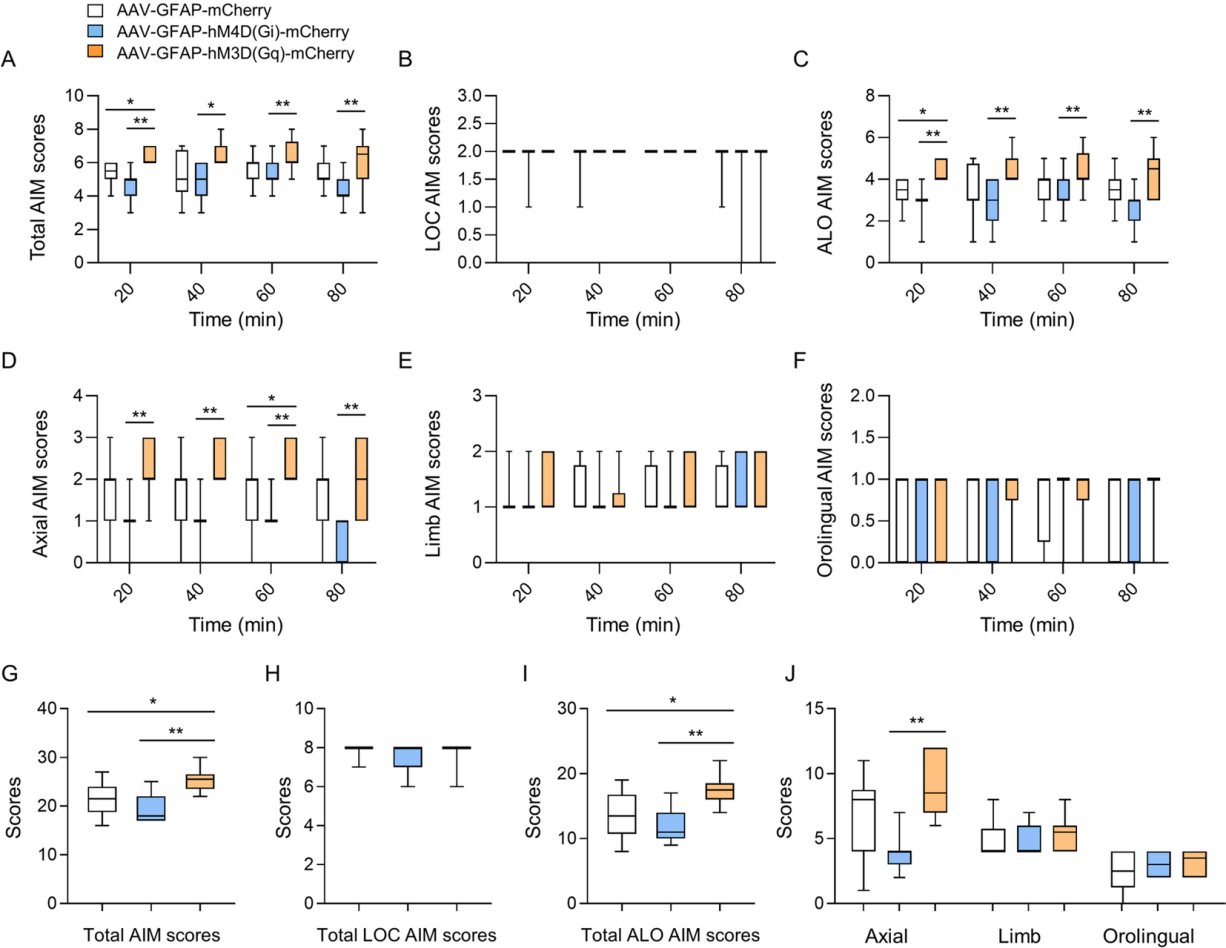
Effects of enhancing and inhibiting astrocytic activity in the dorsal striatum on LID development in the 6-OHDA mouse model. (**A**) The total (ALO + LCO) AIM was scored every 20 min for 80 min on the fifth day of CNO and L-dopa administration. (**B**–**F**) The LOC (**B**), ALO (**C**), axial (**D**), limb (**E**), and orofacial (**F**) AIM behaivors were scored every 20 min for 80 min on the fifth of CNO and L-dopa administration. (**G**) The total (LOC + ALO) AIM scores. (**H**–**J**) The total LOC (**H**), ALO (**I**), axial, limb, and orofacial (J) AIM scores. AAV8-GFAP-mCherry, n = 12; AAV8-hM4D(Gi)-mCherry, n = 11; AAV8-hM3D(Gq)-mCherry, n = 10. *p < 0.05 and **p < 0.01, between the indicated groups (Kruskal–Wallis test, followed by Dunn’s multiple comparisons test). Box-and-whisker plots were presented with the median (horizontal bar) as the central measure, the box representing the interquartile range (25th to 75th percentiles), and the whiskers indicating the minimum and maximum values.

**Fig. 4:**
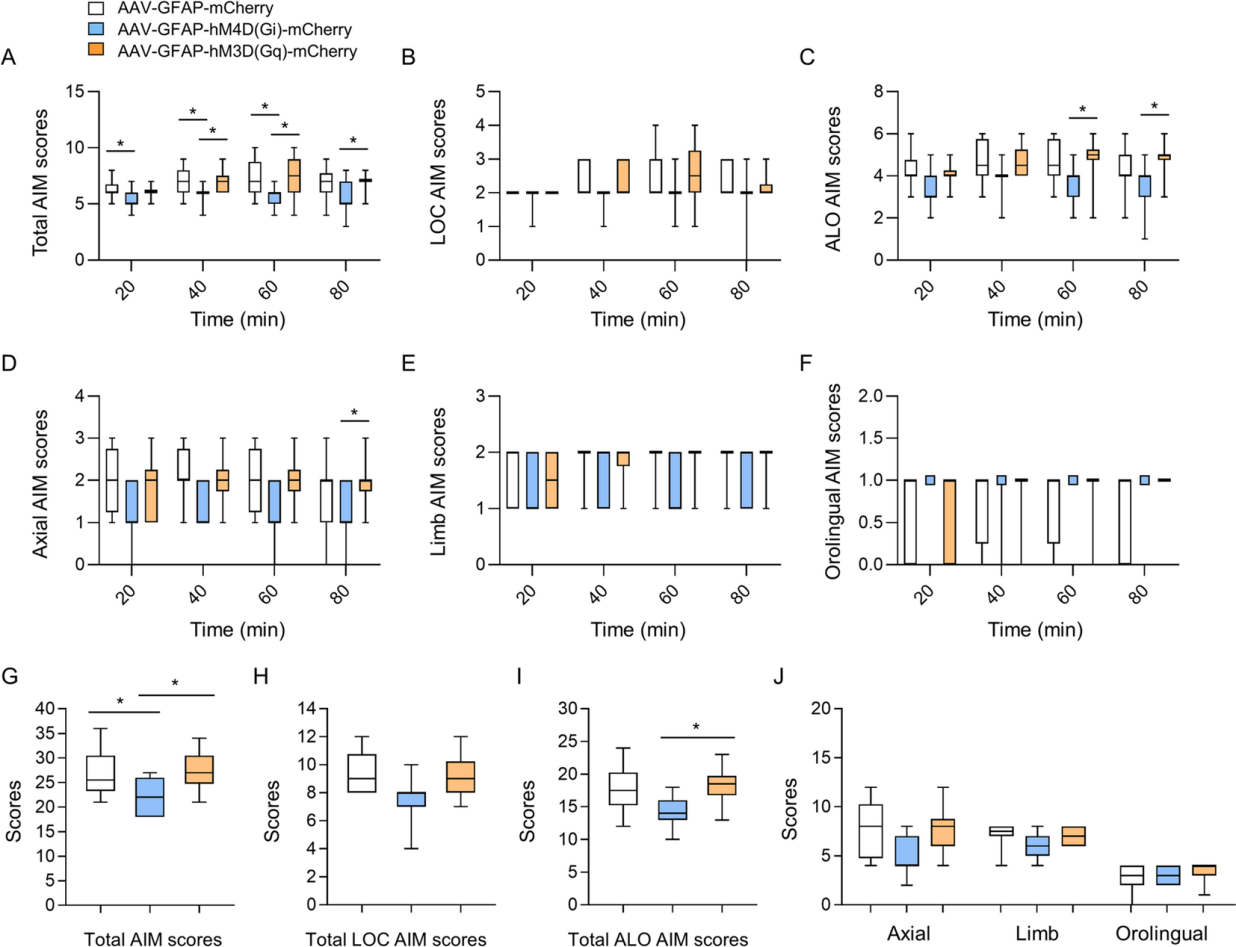
Effects of enhancing and inhibiting astrocytic activity in the dorsal striatum on LID severity in the 6-OHDA mouse model. (**A**) The total (ALO + LOC) AIM was scored every 20 min for 80 min on the tenth day of CNO and L-dopa administration. (**B**–**F**) The total LOC (**B**), ALO (**C**), axial (**D**), limb (**E**), and orofacial (**F**) AIM behaviors were scored every 20 min for 80 min on the tenth day of CNO and L-dopa administration. (**G**) The total (LOC + ALO) AIM scores. (**H**–**J**) The LOC (**H**), ALO (**I**), axial, limb, and orofacial (J) AIM scores. AAV8-GFAP-mCherry, n = 12; AAV8-hM4D(Gi)-mCherry, n = 11; AAV8-hM3D(Gq)-mCherry, n = 10. *p < 0.05, between the indicated groups (Kruskal–Wallis test, followed by Dunn’s multiple comparisons test). Box-and-whisker plots were presented with the median (horizontal bar) as the central measure, the box representing the interquartile range (25th to 75th percentiles), and the whiskers indicating the minimum and maximum values.

GraphPad PRISM (Version 8.4.3, GraphPad Software, San Diego, CA, USA) was used to perform the statistical analyses. The comparison of % right forelimb use in Fig. 2B was performed using an unpaired Student’s t-test. Comparisons of the occupied area and % loss of TH-positive cells in Figs. 1C,D, and 2D were performed using one-way analysis of variance (ANOVA) followed by Tukey’s multiple comparisons test. In Figs. 3 and 4, the significance of AIM scores was determined using the non-parametric Kruskal–Wallis test, followed by Dunn’s multiple comparisons test. In Figs. 1 and 2, data are presented as the mean ± standard deviation (SD). In Figs. 3 and 4, data are presented as the median (horizontal bar), with the interquartile range (25th and 75th percentiles) shown as a box, and the minimum and maximum values indicated by whiskers. Statistical differences were considered significant at the 5% level unless otherwise indicated.

## Results

### Repeated L-dopa administration in the 6-OHDA lesioned model increases astrocytic activity

A 6-OHDA-induced PD mouse model was used to investigate the effects of dopamine depletion and L-dopa administration on astrocyte activation in the dorsal striatum. Only mice with at least 80% reduction in TH + cells in the lesioned SNc (left side) relative to the unlesioned side (right side) were included in the analysis (Fig. 1D). In the three groups with dopamine depletion, the percentages of TH + cells loss were 92.00 ± 1.32%, 89.70 ± 1.54% and 87.51 ± 1.54%, respectively (Fig. 1D). Similar levels of dopaminergic cell death were observed in the 6-OHDA, 6-OHDA + L-dopa (single), and 6-OHDA + L-dopa (repeat) groups (Fig. 1D, *p* > 0.05). The area immunostained by GFAP was significantly increased on the left side (6-OHDA lesioned side) of the striatum in all three 6-OHDA-induced groups compared to that in the control group (Fig. 1B and C, *p* < 0.01). It also increased in the area opposite the 6-OHDA injection (Fig. 1B and C, *p* < 0.05). The occupied area was significantly increased by repeated treatment with L-dopa compared to that in the 6-OHDA-only group (Fig. 1B and C, *p* < 0.05). In addition, the area occupied by the 6-OHDA lesioned side was significantly increased in the repeated L-dopa administration group compared to that in the single L-dopa administration group (Fig. 1C, *p* < 0.01). These results indicate that long-term L-dopa treatment in PD increases astrocyte activation.

### AAV8-mediated mCherry expression is induced in the striatal astrocytes of the 6-OHDA-lesion model

AAV-GFAP-mCherry, AAV-hM4D(Gi)-mCherry, and AAV-hM3D(Gq)-mCherry were injected into the dorsal striatum, and 6-OHDA was injected into the SNc of mice to investigate the regulatory effects of astrocytic activation or inhibition on LID development using the Gq- or Gi-coupled DREADD system (Fig. 2A). L-dopa administration led to a significant increase in the percentage of right forelimb use across all three experimental groups (Fig. 2B, *p* < 0.01), indicating the therapeutic effect of L-dopa. Only mice with at least 80% reduction in TH + cells in the 6-OHDA lesioned SNc relative to the unlesioned side were included in the final analysis (Fig. 2C and D). In the three groups with dopamine depletion, the percentages of loss of TH + cells were 93.43 ± 1.93%, 91.43 ± 2.35%, and 92.25 ± 1.55%, respectively (Fig. 2D). Similar levels of dopaminergic cell death were observed in AAV-GFAP-mCherry, AAV-hM4D(Gi)-mCherry, and AAV-hM3D(Gq)-mCherry groups (Fig. 2D, *p* > 0.05). The percentages of mCherry expression in the dorsal striatum (DS) region following viral injection were 45.9 ± 3.6%, 49.9 ± 3.2%, and 44.7 ± 2.3% for groups AAV-GFAP-mCherry, AAV-hM4D(Gi)-mCherry, and AAV-hM3D(Gq)-mCherry groups, respectively (Fig. 2E). Its co-localization with GFAP was examined in the dorsal striatum to confirm virus-mediated mCherry expression (Fig. 2F). Following AAV-GFAP-mCherry, AAV-hM4D (Gi)-mCherry, and AAV-hM3D (Gq)-mCherry injection, mCherry was co-localized in the injected area of the brain (Fig. 2F). In contrast, it did not co-localize with neurons (Fig. 2G).

### CNO-dependent activation of hM3D(Gq)-mCherry expressing striatal astrocytes increases LID development in the 6-OHDA mouse model

On the fifth day after L-dopa injection, in the early stage of LID development, stimulation with AAV-GFAP-hM3D(Gq)-mCherry significantly increased the total AIM score compared to that in the AAV-GFAP-mCherry and AAV-GFAP-hM4D(Gi)-mCherry groups (Fig. 3A and G). In particular, the ALO AIM score, which is similar to the behavioral pattern observed in patients with LID, increased over 80 min compared to the AAV-GFAP-mCherry and AAV-GFAP-hM4D (Gi)-mCherry groups (Fig. 3C and I). Among the three ALO scores, the axial score increased in the AAV-GFAP-hM3D(Gq)-mCherry group, whereas no differences were observed in the limb and orolingual scores (Fig. 3D–F, and J). No difference was observed in the LOC score of astrocytic-DREADD mice (Fig. 3B and H). Stimulation with astrocytic Gi-DREADD slightly decreased the total AIM and ALO scores compared to the control; however, the decrease was not significant. These results suggest that L-dopa administration, along with the induction of astrocytic Gq-DREADD activation in the striatum, may promote LID development.

### CNO-dependent activation of hM3D(Gi)-mCherry expressing striatal astrocytes decreases the severity of LID in the 6-OHDA mouse model

An AIMS test was performed on day 10 after L-dopa and CNO injections to determine the effects of astrocytic activation on LID progression. Stimulation of astrocytic Gi-DREADD through CNO injection significantly decreased the total AIM scores compared to the AAV-GFAP-mCherry group (Fig. 4A and G). Stimulation of astrocytic Gi-DREADD through CNO injection significantly decreased the ALO scores compared to the AAV-GFAP-hM3D(Gq)-mCherry group (Fig. 4C and I). However, stimulation of astrocytic Gq-DREADD using CNO did not cause a significant difference in the total AIM, LOC, and ALO scores compared to the AAV-GFAP-mCherry group (Fig. 4G–J). It did not significantly decrease LOC scores (Fig. 4B and H). Similar to the results in Fig. 3, stimulation of astrocytic Gi-DREADD with CNO tended to reduce the axial score at 80 min after L-DOPA administration compared to the AAV-GFAP-hM3D(Gq)-mCherry group. However, no significant reduction was observed in the limb and orolingual scores (Fig. 4D–F, and J). These results suggest that astrocytic Gq-DREADD stimulation affects the mid-treatment phase progression of LID. In contrast, astrocytic Gi-DREADD stimulation through CNO injection had little effect on early LID development but showed an inhibitory effect on the worsening of ongoing LID.

## Discussion

Astrocytes play a pivotal role in the pathogenesis of PD^33,34^. Reactive astrocytes are not only abundant in the brains of patients with PD but are also associated with the severity of LID development^17,33^. Reactive astrogliosis, characterized by increased expression of GFAP and astrocyte extension, has been reported in PD animal models^35^. In this study, a 6-OHDA-induced PD mouse model was generated using a DREADD system modulated by a GFAP-specific promoter.

The dorsal striatum plays a central role in LID pathophysiology^30,36–39^. In the dorsal striatum, astrocytes regulate specific synapses in medium-sized spiny neurons via glutamate receptor activation^40^. L-dopa-induced dyskinesia involves both presynaptic and postsynaptic dysfunctions of dopaminergic transmission influenced by non-dopaminergic systems^18,41^. Consistent with previous studies^15,42–44^, this study demonstrated that repeated L-dopa administration can induce astrocytic hyperactivation. Dopamine depletion induced by the 6-OHDA-lesion triggered astrocyte hyperactivation, which was exacerbated by repeated L-dopa treatment. Therefore, astrocytes may participate in striatal dysfunction, which is a pathway underlying the motor deficits in PD^45^. Furthermore, astrocytes contribute to the excessive production of toxic molecules, including nitric oxide and cytokines^46^. LID development is accompanied by the upregulation of an inflammatory cascade involving nitric oxide^15^. Elevated nitric oxide synthase and cyclooxygenase-2 have been observed in the SNc and striatum in the brain of a postmortem patient with PD^47,48^. Chronic administration of L-dopa in PD has been shown to induce blood–brain barrier hyperpermeability and angiogenesis^49–51^. A perivascular recruitment of hypertrophic astrocytic processes following chronic L-DOPA has been reported^51^. Upregulation of the vascular endothelial growth factor (VEGF), a key mediator of these vascular changes, has been implicated in the development of dyskinesia in the PD, and inhibition of the VEGF signaling attenuated the development of LID^49^. As shown in Fig. 1B, astrocytes surrounding the blood vessels in the 6-OHDA lesioned striatum were strongly stained. Both the staining intensity and area of GFAP were increased by chronic L-dopa treatment. While L-dopa administration appears to enhance astrocytic activation and signal intensity around blood vessels, this study did not specifically analyze astrocytes in close proximity to cerebral vasculature. Further studies are needed to verify these findings by performing vascular-specific staining along with GFAP staining in the brain tissue.

The DREADD system, a chemogenetic tool engineered to control GPCR signaling pathways^19^, is suitable for modulating astrocytic activity, which is highly responsive to GPCR signaling^52,53^. CNO is known to be metabolized into clozapine in vivo, which can interact with 5-HT2A and dopamine D2/3 receptors, potentially leading to off-target effects^54^. The fact that CNO is neurochemically active highlights the need for caution in assigning its effects solely to DREADD mechanisms. Although a high dose of CNO (20 mg/kg) resulted in detectable levels of clozapine in plasma^55^, clozapine was not detected in plasma following a low dose of CNO (1 mg/kg)^56^. Recently, activation of astrocytic hM3D(Gq) and hM4D(Gi) pathways via chemogenetics using CNO has been reported^23,24,54,55^. Chemogenetic modulation of striatal astrocytes improves open-field exploratory behavior and asymmetrical motor deficits in PD mice^56^. In this study, AAV8-mediated mCherry expression in striatal astrocytes co-localized with GFAP-positive cells but not with NeuN-positive cells. The area of AAV-GFAP-DREADD-mCherry expression in the striatum did not fully cover the entire area occupied by striatal astrocytes; however, a significant overlap with astrocytes was observed in the area where GFAP-DREADD-mCherry was expressed. Compared with the control group, the GFAP-Gi-mCherry group exhibited fewer morphological branches, whereas the GFAP-Gq-mCherry group was more widely spread. The area of AAV-GFAP-DREADD-mCherry expression in the striatum did not fully cover the entire area occupied by striatal astrocytes. However, astrocytic activity modulation by local infusion of AAV8 into the dopamine-denervated striatum and systemic administration of CNO induced behavioral changes in LID in mice. The induction of astrocyte activation by AAV-GFAP-Gq-DREADD appeared to have a greater effect during mid-treatment phase progression, whereas the induction of astrocyte inhibition by AAV-GFAP-Gi-DREADD influenced later stages. Repeating the experiment and increasing the frequency of measurements from the first day could provide more detailed conclusions about the study results.

L-dopa therapy affects both neuronal and glial compartments within the basal ganglia and midbrain^13,18^, and recent studies have reported on the effects of LID on astrocytic activity^15,16,28^. For example, L-dopa accumulation has been reported in astrocytic endfeet surrounding the blood vessels and astrocyte cell bodies^57^. Inhibiting astrocytic activity using a nitric oxide synthase inhibitor significantly suppressed AIMs in unilaterally 6-OHDA-lesioned rats^15^. Arundic acid (known as ONO-2506), an astrocyte regulator, specifically inhibits S100β protein in astrocytes^58^. ONO-2506 mitigates LID severity by enhancing glutamate transporter-1 expression and glutamate uptake^16^. Furthermore, astrocytes alter synaptic plasticity and phosphorylation of the NMDA receptor signaling pathway by affecting NMDA receptor overexpression and accelerating LID development^59^. A previous study assessing the uptake, metabolism, and L-dopa and dopamine release in striatal astrocytes demonstrated that striatal astrocytes can take up dopamine, L-dopa, and their metabolites and that the intracellular L-dopa level in these astrocytes decreases rapidly^60^. In this study, although L-dopa was injected combined with CNO, the initial pharmacological effect of L-dopa was observed in the cylinder test. Although it is well known that PD involves specific pathological mechanisms, astrocytes are also found to be essential for maintaining neuronal health and brain homeostasis^61^. Astrocytes are known to produce neurotrophic factors such as glial cell line-derived neurotrophic factor, that promote proliferation and integrity of dopaminergic cells^62^. It is necessary to consider the potential implications or consequences associated with reducing astrocytic involvement in the PD brain. Therefore, research utilizing the DREADD approach warrants careful consideration and the development of targeted strategies that selectively suppress pathological processes while preserving beneficial astrocytic functions.

In conclusion, regulating astrocytic activity in the dorsal striatum could be a potential strategy for controlling LID in PD. It provides novel insights into the role of astrocytes in LID and proposes potential therapeutic strategies for its mitigation. The results of this study demonstrate that AAV-mediated gene regulation enables region-specific control of astrocyte activity in the brain. This study is the first to directly modulate astrocytic activity in the context of LID, thereby elucidating their role in the pathophysiology of this complication. Moreover, our research highlights the need for pharmacological approaches to target astrocytes systemically, as their invasiveness and technical demands limit current viral delivery methods, such as the inability to cover the entire striatal region and risk factors associated with the use of surgical techniques. Lastly, further research is needed to elucidate the mechanisms by which activating and inhibiting astrocytic activity alters LID.

## Data Availability

The datasets used and analyzed during the current study are available from the corresponding author on reasonable request. *Corresponding author: Kyoung-Shim Kim, Ph.D. Laboratory Animal Resource Center, Korea Research Institute of Bioscience and Biotechnology (KRIBB), Daejeon 34,141, Republic of Korea, Tel: + 82-42-860–4634, E-mail: kskim@kribb.re.kr.

## Abbreviations

6-OHDA: 6-Hydroxydopamine
AIM: Abnormal involuntary movement
AAVs: Adeno-associated viruses
CNO: Clozapine N-oxide
DREADDs: Designer receptors exclusively activated by designer drugs
GPCRs: G-protein-coupled receptors
hM1D(Gq): Human M1 muscarinic acetylcholine receptor DREADD with Gq signaling
hM2D(Gi): Human M2 muscarinic acetylcholine receptor DREADD with Gi signaling
hM3D(Gq): Human M3 muscarinic acetylcholine receptor DREADD with Gq signaling
hM4D(Gi): Human M4 muscarinic acetylcholine receptor DREADD with Gi signaling
hM5D(Gq): Human M5 muscarinic acetylcholine receptor DREADD with Gq signaling
GFAP: Glial fibrillary acidic protein
IHC: Immunohistochemistry
L-dopa: 3,4-Dihydroxy-L-phenylalanine
LID: L-dopa-induced dyskinesia
PBS: Phosphate-buffered saline
PD: Parkinson’s disease
SNc: Substantia nigra
ST: Striatum
TBS: Tris-buffered saline
TH: Tyrosine hydroxylase
